# Prevalencia de enfermedades no transmisibles y factores de riesgo en población rural de San Luis, Argentina

**DOI:** 10.31053/1853.0605.v81.n1.42138

**Published:** 2024-03-27

**Authors:** Eloy Salinas, María Cecilia De Pauw, María Fernanda Aguirre, María Jimena Marro, Christian Ballejo, Alejandro Félix Sturniolo, Alicia EB Lawrynowicz

**Affiliations:** 1 Universidad Nacional de San Luis. Facultad de Química Bioquímica y Farmacia; 2 Universidad Nacional de San Luis; 3 Instituto Nacional de Epidemiología "Dr. Juan H. Jara"; 4 Universidad Nacional de San Luis. Facultad de Química Bioquímica y Farmacia. Cátedra de análisis

**Keywords:** enfermedades no transmisibles, salud rural, epidemiología, Argentina, noncommunicable diseases, rural health, epidemiology, Argentina, doenças não transmissíveis, saúde rural, epidemiologia, Argentina

## Abstract

El objetivo de este estudio fue estimar la prevalencia de diabetes mellitus (DM) y factores de riesgo cardiovascular en una población rural de la provincia de San Luis, Argentina. Estudio transversal desarrollado entre septiembre y noviembre de 2017 con habitantes de 18 años y más de cuatro localidades del departamento Juan Martín de Pueyrredón, San Luis. Los participantes respondieron preguntas por autorreporte sobre aspectos sociodemográficos, hábitos, factores psicosociales y de riesgo para enfermedades no transmisibles; se realizaron mediciones físicas, cuestionario FINDRISC y extracción de muestras de sangre. Se obtuvieron estimaciones univariadas estratificadas por sexo con su intervalo de confianza del 95% (IC95%). Se trabajó con factores de expansión de la muestra; se calcularon prevalencias crudas y ajustadas por edad. La población estuvo constituida por 424 varones (52,5%, IC95%: 46,0-58,9) y 384 mujeres (47,5%, IC95%: 41,1-54,0). Las prevalencias ajustadas por edad para ambos sexos (por autorreporte) fueron: DM 11,8% (IC95%: 8,2-15,4); hipertensión arterial (HTA): 35,5% (IC95%: 31,0-40,1); colesterol elevado: 20,3% (IC 16,0-24,7). Los varones tuvieron colesterol HDL deseable y tensión arterial elevada en una proporción significativamente superior a las mujeres; las mujeres obesidad abdominal en mayor magnitud. El 16,4 % (IC95%: 11,0 - 23,6) ostentó riesgo alto-muy alto de desarrollar DM tipo 2 en los próximos 10 años. Las prevalencias ajustadas por edad de DM, HTA y colesterol elevado fueron inferiores a la de la población urbana de la provincia de San Luis. Destacamos la contribución pionera de este trabajo al conocimiento del perfil de salud de las comunidades rurales de Argentina

## Abstract (en)

The objective of this study was to estimate the prevalence of diabetes mellitus (DM) and cardiovascular risk factors in a rural population in the province of San Luis, Argentina. Cross-sectional study developed between September and November 2017 with 18-year-old inhabitants and more than four towns in the Juan Martín de Pueyrredón department, San Luis. The participants answered questions by self-report on sociodemographic aspects, habits, psychosocial and risk factors for non-communicable diseases; physical measurements, FINDRISC questionnaire and blood sample extraction were performed. Univariate estimates stratified by sex with their 95% confidence interval (95%CI) were obtained. We worked with sample expansion factors; crude and age-standardized prevalence were calculated. The population consisted of 424 men (52.5%, 95%CI: 46.0-58.9) and 384 women (47.5%, 95%CI: 41.1-54.0). The age-standardized prevalence for both sexes (by self-report) were: DM 11.8% (95%CI: 8.2-15.4); arterial hypertension (AHT): 35.5% (95% CI: 31.0-40.1); high cholesterol: 20.3% (CI 16.0-24.7). Males had significantly higher desirable HDL cholesterol and elevated blood pressure than females; women abdominal obesity in greater magnitude. 16.4% (95% CI: 11.0 - 23.6) had a high-very high risk of developing type 2 DM in the next 10 years. The age-standardized prevalence of DM, hypertension, and high cholesterol were lower than those of the urban population of the province of San Luis. We highlight the pioneering contribution of this work to the knowledge of the health profile of rural communities in Argentina

## Resumo

O objetivo deste estudo foi estimar a prevalência de diabetes mellitus (DM) e fatores de risco cardiovascular em uma população rural da província de San Luis, Argentina. Estudo transversal desenvolvido entre setembro e novembro de 2017 com habitantes de 18 anos e mais de quatro cidades do departamento de Juan Martín de Pueyrredón, San Luis. Os participantes responderam questões por autorrelato sobre aspectos sociodemográficos, hábitos, psicossociais e fatores de risco para doenças não transmissíveis; medidas físicas, questionário FINDRISC e coleta de amostra de sangue foram realizadas. Foram obtidas estimativas univariadas estratificadas por sexo com seu intervalo de confiança de 95% (IC95%). Trabalhamos com fatores de expansão amostral; prevalências brutas e ajustadas por idade foram calculadas. A população foi composta por 424 homens (52,5%, IC95%: 46,0-58,9) e 384 mulheres (47,5%, IC95%: 41,1-54,0). As prevalências ajustadas por idade para ambos os sexos (por autorrelato) foram: DM 11,8% (IC95%: 8,2-15,4); hipertensão arterial (HAT): 35,5% (IC 95%: 31,0-40,1); colesterol alto: 20,3% (IC 16,0-24,7). Os machos tinham colesterol HDL desejável significativamente mais alto e pressão arterial elevada do que as fêmeas; mulheres com obesidade abdominal em maior magnitude. 16,4% (95% CI: 11,0 - 23,6) tinham um risco alto-muito alto de desenvolver DM tipo 2 nos próximos 10 anos. As prevalências ajustadas por idade de DM, hipertensão e colesterol alto foram menores do que as da população urbana da província de San Luis. Destacamos a contribuição pioneira deste trabalho para o conhecimento do perfil de saúde das comunidades rurais da Argentina

**CONCEPTOS CLAVE**

**Qué se sabe sobre el tema.**

Existe un déficit de información epidemiológica sobre el perfil de enfermedades no transmisibles (ENT) en áreas rurales de la República Argentina. Las encuestas nacionales de factores de riesgo (ENFR) llevadas a cabo en poblaciones de más de 5000 habitantes durante los años 2005, 2009 y 2013 obtuvieron información por autorreporte mientras que la del año 2018 incorporó mediciones físicas y determinaciones bioquímicas.

**Qué aporta este trabajo.**

Este trabajo reseña los resultados del primer estudio epidemiológico sobre ENT llevado a cabo en las poblaciones rurales del departamento Juan Martín de Pueyrredón de la Provincia de San Luis, Argentina. El propósito del mismo es contribuir a la planificación de políticas de promoción y prevención de la salud en función de las características de las comunidades.

**Divulgación**

En este trabajo se detallan los resultados del primer estudio epidemiológico multidisciplinario sobre los determinantes sociales, estilos de vida. síndrome metabólico y factores de riesgo cardiovascular, realizado en poblaciones rurales del departamento Juan Martín de Pueyrredón de la provincia de San Luis, Argentina. El propósito de este trabajo es contribuir a la planificación de políticas de promoción y prevención de la salud en función de las características de las comunidades rurales.

## Introducción

Las enfermedades no transmisibles (ENT) dan cuenta de más del 70% del total de muertes a nivel global. Este grupo, integrado por las enfermedades cardiovasculares, el cáncer, la diabetes mellitus (DM) y las enfermedades respiratorias crónicas, comparten factores de riesgo relacionados con comportamientos y hábitos en ocasiones modificables^1^. En la Región de las Américas, en 2019, el 81% de las muertes fueron a causa de ENT, representando 121 millones de años de vida perdidos por muertes prematuras, 226 millones de años de vida ajustados por discapacidad y 105 millones de años vividos con discapacidad^2^. En Argentina, en consonancia con lo que ocurre en la región y el mundo, el 78% de las muertes ocurridas en 2020 fueron por ENT^1^.

La información sobre las ENT en Argentina proviene de encuestas nacionales de base poblacional desarrolladas en áreas urbanas^3^, lo que se traduce en un desconocimiento del comportamiento de estas enfermedades en la población rural. Por otro lado, investigaciones llevadas a cabo en comunidades rurales de diversos países subrayan las dificultades para la accesibilidad al sistema de salud; aspectos como la localización geográfica de las poblaciones, la limitación de recursos de transporte e infraestructura y la marginación del desarrollo socioeconómico las ubican en situación de vulnerabilidad socio económica y epidemiológica^4^. A su vez, con relación a los hábitos de consumo alimentario, un estudio desarrollado en Argentina encontró un mayor consumo aparente de calorías, harina de trigo, margarina, grasas animales, azúcar de mesa y sal en la población rural, acompañado de un menor consumo de frutas y hortalizas, lo que podría indicar la existencia de vulnerabilidad alimentaria^5^.

Estos indicios levantan la preocupación por las condiciones de salud de la población rural. Dada la vacancia de conocimiento en esta temática, el objetivo de esta investigación fue estimar la prevalencia de DM y factores de riesgo cardiovascular en una población rural del departamento Juan Martín de Pueyrredón, San Luis, Argentina, en el año 2017.

## Métodos

Este estudio de corte transversal fue desarrollado en un área rural de la provincia de San Luis, Argentina, entre septiembre y noviembre del año 2017. La población estuvo conformada por los habitantes de las localidades de Beazley, Zanjitas, Alto Pelado y Cazador del departamento Juan Martín de Pueyrredón de la provincia de San Luis. Se incluyeron participantes de 18 años o más con un tiempo mínimo de residencia en el lugar de 6 meses, que se encontraran en el hogar y aceptaran participar del estudio. Fueron excluidas aquellas personas en tratamiento crónico con corticoides sistémicos, con antecedente de hospitalización en el mes previo al inicio del estudio y mujeres embarazadas. Se invitó a participar a todas las personas que cumplían con los criterios de inclusión.

En el diseño del estudio, no se estableció una muestra a priori ya que la intención fue incluir a toda la población del área rural. Se visitaron todas las viviendas del área de estudio y se invitó a participar a las personas que cumplieran con los criterios de inclusión y exclusión. No fue posible, por cuestiones de inaccesibilidad geográfica, llegar a unas pocas viviendas. Luego, la muestra obtenida se corrigió en función de la tasa de respuesta, con base en el marco muestral construido a partir de la cartografía digital y los datos de la población del área de estudio, de fuente censal.

La investigación consistió en dos fases; durante la primera los participantes respondieron un cuestionario con preguntas previamente validadas sobre aspectos sociodemográficos, hábitos, factores psicosociales y factores de riesgo para ENT, por autorreporte. La segunda etapa incluyó mediciones físicas, cuestionario FINDRISC y extracción de muestras de sangre; para mayores detalles sobre la metodología puede consultarse una publicación previa relacionada con este trabajo^6^. Para estimar el riesgo de desarrollar DM tipo 2 (DM2) a 10 años se tomó como referencia el test FINDRISC^7^, que fue realizado a quienes autorreportaron no tener antecedentes de DM. El riesgo de desarrollar DM2 se estableció según el puntaje asignado al*score*en cada una de las dimensiones^3^.

Se expandió la muestra obtenida en primera y segunda etapa a partir de la estructura de la población del último Censo Nacional de Población, Hogares y Viviendas del año 2010^8^, estratificada por sexo y edad. El tamaño de muestra total de la primera etapa (n=374) se obtuvo considerando una prevalencia de 36% para hipertensión arterial (HTA), intervalos de confianza del 95% (IC95%) y un efecto de diseño de 2,5 que define una precisión del 7%; el resultado de la expansión se observa en la Tabla 1.

**Tabla Nº 1:** Expansión de la muestra según localidad de procedencia para ambas etapas, población de ambos sexos de 18 años y más. Departamento Juan M. de Pueyrredón, San Luis, 2017

**Localidad**	**Muestra obtenida 1era etapa**	**Población expandida e IC 95%**	**Error estándar**	**CV**
Alto Pelado	68	76 (67-85)	4,48	0,06
Beazley	192	564 (514-614)	25,5	0,04
Cazador	34	42 (18-66)	12,35	0,29 ^a^
Zanjitas	80	126 (107-145)	9,5	0,07
Total	374	808 (749-867)	30,2	0,04
**Localidad**	**Muestra obtenida 2da. etapa**	**Población expandida e IC 95%**	**Error estándar**	**CV**
Alto Pelado	53	76 (67-85)	4,49	0,06
Beazley	112	564 (481-647)	42,1	0,07
Cazador	25	42 (17-67)	12,8	0,30 ^a^
Zanjitas	61	126 (94-157)	16	0,13
Total	251	808 (723-893)	43	0,05

^a^ La estimación debe ser considerada con precaución por tener un CV entre 20 % y 30 %.

En la primera etapa, las variables construidas y sus categorías fueron: grupo etario (18-24, 25-34, 35-49, 50-64, 65 años y más), situación conyugal (solo/a: personas separadas, divorciadas, viudas o solteras; en pareja: en unión o casadas), educación (nunca asistió: nunca asistieron a un establecimiento educativo; asiste o asistió: asisten o asistieron a un establecimiento educativo), nivel educativo (inferior: asistieron a un establecimiento educativo sin completar el nivel secundario; superior: completaron el nivel secundario, iniciaron o terminaron el nivel terciario, universitario o posgrado), situación laboral (sin trabajo: jubilado o pensionado, no quería, no deseaba o no podía trabajar, no tenía o no conseguía trabajo; con trabajo: trabajó al menos una hora la semana anterior), cobertura de salud (sí: tienen obra social, plan de salud privado o mutual; no: sin obra social o prepaga, eventualmente con servicio de emergencia o plan estatal de salud), tabaquismo (fuma actualmente, fumó alguna vez, nunca fumó), consumo de frutas o verduras (bajo: consumen frutas o verduras 6 días a la semana o menos; alto: consumen frutas o verduras los 7 días de la semana), antecedentes familiares de DM (sí: antecedentes de madre, padre o hermano/a o abuelo/a; no: sin antecedentes en familiares), prevalencia de DM, HTA o colesterol elevado (sí: autorreportaron tener HTA, DM o colesterol elevado, respectivamente; no: respuesta negativa), antecedentes cardiovasculares (sí: sufrieron evento cardíaco como infarto agudo de miocardio, angina de pecho, crisis cardíaca u otra enfermedad cardíaca; no: no sufrieron evento cardíaco).

Las variables de la segunda etapa [tensión arterial diastólica (TAD), tensión arterial sistólica (TAS), IMC, perímetro de cintura, glucemia basal, hemoglobina glicosilada (HbA1c), colesterol total, HDL y LDL] fueron operacionalizadas siguiendo recomendaciones internacionales en la materia^9^.

Se obtuvieron estimaciones univariadas y estratificadas por sexo con su IC95% y el coeficiente de variación (CV); para el cálculo del IC se ajustó un modelo de regresión logística, obteniendo un intervalo de tipo Wald en la escala de probabilidades logarítmicas que luego se transformó en la escala de probabilidad. Se tuvieron en cuenta criterios de calidad a partir de los CV. Se compararon estimaciones por sexo mediante test de Chi cuadrado con ajuste de Rao & Scott^9^ para variables categóricas.

Para los cálculos de prevalencia de DM, HTA o colesterol elevado por autoreporte, se tomó como casos positivos a quienes refirieron tener el evento; los que respondieron de manera negativa o "no sabe/no contesta" se consideraron negativos. Se calculó además la prevalencia combinada de cada variable sumando a los casos positivos por autorreporte aquellos que superaron los puntos de corte de normalidad en la etapa de determinaciones bioquímicas.

Se calcularon prevalencias ajustadas por edad por método directo para DM, HTA y colesterol elevado, con un IC95%. Como población estándar se utilizó la estimada para Argentina por edad y sexo en 2017^10^.

El procesamiento y análisis de los datos se realizó en lenguaje R 4.2.2^11^, ejecutado en entorno RStudio 2022.07.2. Se utilizaron paquetes específicos tales como *tidyverse*, *survey* y *srvyr*. El protocolo de investigación fue aprobado por el Comité de Ética en Investigación del Instituto Nacional de Epidemiología "Dr. Juan H. Jara", que está acreditado por el Comité de Ética Central, bajo el número 059/2016 y se encuentra registrado en el Registro Nacional de Investigaciones en Salud (RENIS), código: CE00264.

## Resultados

La población estuvo constituida por 424 varones (52,5%, IC95%: 46,0-58,9) y 384 mujeres (47,5%, IC95%: 41,1-54,0). Las mujeres tuvieron nivel educativo superior, se encontraron sin trabajo, con bajo nivel de actividad física y fueron no fumadoras en una proporción significativamente mayor a los varones (Tabla 2).

**Tabla Nº 2:** Distribución de variables sociodemográficas y hábitos en población de 18 años y más según sexo, zona rural del Departamento Juan M. de Pueyrredón, San Luis, 2017 (N=808).

**Variables**	**Ambos sexos (N = 808)**		**Mujeres (N = 384)**		**Varones (N = 424)**		p valor ^*^
	**Frec.**	**Porcentaje (IC95%)**	**Frec.**	**Porcentaje (IC95%)**	**Frec.**	**Porcentaje (IC95%)**	
	**abs.**		**abs.**		**abs.**		
**Rango de edad**							0,723
18-24	166	20,5 (14,8-27,7)	80	20,8 (15,4-27,6)	86	20,3 (11,4-33,4) ^a^	
25-34	196	24,3 (18,9-30,5)	105	27,3 (20,4-35,6)	91	21,5 (14,1-31,4)	
35-49	187	23,1 (18,3-28,8)	77	20,1 (15,4-25,6)	110	25,9 (18,1-35,8)	
50-64	150	18,6 (14,4-23,6)	68	17,7 (12,3-24,9)	82	19,3 (13,5-26,8)	
65 y más	109	13,5 (10,5-17,2)	54	14,1 (10,1-19,2)	55	13,0 (8,8-18,7)	
**Situación conyugal**							0,204
Solo/a	322	39,8 (33,5-46,5)	133	34,6 (27,8-42,1)	189	44,6 (34,4-55,2)	
En pareja	480	59,4 (52,8-65,8)	247	64,4 (56,9-71,3)	233	54,9 (44,3-65,1)	
Sin dato	6	/// ^b^	4	/// ^b^	2	/// ^b^	
Educación							0,31
Nunca asistió	39	4,9 (2,9-8,0) ^a^	19	5,0 (2,8-8,5) ^a^	20	/// ^b^	
Asiste o asistió	763	94,4 (91,1-96,5)	359	93,5 (89,2-96,2)	404	95,2 (89,3-97,9)	
Sin dato	6	/// ^b^	6	/// ^b^	-	-	
**Nivel educativo**							< 0,05
Inferior	511	63,2 (56,9-69,1)	210	54,7 (47,1-62,2)	301	70,9 (60,7-79,3)	
Superior	252	31,2 (25,6-37,4)	149	38,8 (31,7-46,4)	103	24,3 (16,5-34,3)	
Sin dato	45	5,6 (3,5-8,9) ^a^	25	6,5 (3,8-10,8) ^a^	20	/// ^b^	
**Situación laboral**							< 0,01
Sin trabajo ^c^	284	35,1 (29,2-41,5)	174	45,4 (38,0-53,1)	110	25,7 (17,7-35,9)	
Con trabajo	524	64,9 (58,5-70,8)	210	54,6 (46,9-62,0)	314	74,3 (64,1-82,3)	
**Cobertura de salud**							0,79
Si	540	66,8 (60,2-72,8)	264	68,7 (61,0-75,6)	276	65,0 (54,3-74,4)	
No	254	31,5 (25,6-38,0)	114	29,8 (23,0-37,6)	140	33,0 (23,8-43,6)	
Sin dato	14	/// ^b^	6	/// ^b^	8	/// ^b^	
**Actividad física**							< 0,01
Bajo	526	65,1 (58,5-71,2)	288	75,0 (68,0-81,0)	238	56,1 (45,6-66,0)	
Moderado	209	25,9 (20,5-32,1)	74	19,3 (13,9-26,0)	135	31,9 (23,1-42,2)	
Intenso	73	9,0 (5,7-13,9) ^a^	22	5,7 (3,2-10,0)	51	12,0 (6,6-20,9)	
**Tabaquismo**							< 0,01
Fumador actual	239	29,6 (23,4-36,6)	60	15,6 (11,1-21,6)	179	42,2 (32,0-53,1)	
Ex fumador	196	24,3 (19,6-29,7)	89	23,3 (17,6-30,4)	107	25,1 (18,1-33,8)	
No fumador	373	46,1 (39,7-52,6)	235	61,1 (53,4-68,0)	138	32,7 (23,8-43,0)	
**Consumo de frutas y verduras**							0,56
Bajo	391	48,4 (41,9-54,9)	178	46,3 (38,7-53,8)	213	50,3 (40,0-60,6)	
Alto	370	45,8 (39,4-52,4)	189	49,2 (41,7-56,9)	181	42,7 (32,8-53,1)	
Sin dato	47	/// ^b^	17	/// ^b^	30	/// ^b^	
Utiliza para cocinar							0,34
Aceite	788	97,6 (91,9-99,3)	379	98.7 (95,4-99,6)	409	96,6 (84,2-99,3)	
Ns/Nc	20	/// ^b^	5	/// ^b^	15	/// ^b^	
**Uso de sal al cocinar**							0,08
Si	757	93,7 (88,7-96,5)	366	95,3 (91.1-97.7)	391	92,2 (82,6-96,7)	
No	39	4,7 (2,8-8,2) a	17	/// ^b^	22	/// ^b^	
No cocina	12	/// ^b^	1	/// ^b^	11	/// ^b^	
**Uso de sal en mesa**							0,55
Siempre	103	12,7 (8,5-19,1)	41	10,7 (6,9-16,6) ^a^	62	14,7 (7,9-25,9) ^a^	
A veces	136	16,8 (12,7-22,8)	58	15,1 (11,0-22,0)	78	18,4 (11,5-28,2) ^a^	
Nunca	556	68,8 (63,1-75,9)	274	71,3 (66,2-79,5)	282	66,5 (55,7-76,4)	
Sin dato	13	/// ^b^	11	/// ^b^	2	/// ^b^	
^*^ Test X^2^ con ajuste Rao & Scott							
^a^ La estimación debe ser considerada con precaución por tener un CV entre 20 % y 30 %.							

La estimación no es confiable ya que el CV es superior al 30%, la cantidad de casos involucrados es insuficiente o la proporción es menor al 5%.

570 participantes (71%, IC95%: 63,9-77,2) tenían hijos, con una mediana de 3 (IC95%: 3-4). Mayoritariamente nacieron en Argentina [804 (99,5%) IC95%: 98,6-99,9], específicamente en la provincia de San Luis [710 (87,9 %) IC95%: 83,4-91,3]. Respecto de la adscripción étnica, 617 (76,4%, IC95%: 70,9-81,1) refirieron descender de criollos, 83 (10,2%, IC95%: 7,3-14,1) de europeos y 17 (2,1%, IC95%: 1,1-4,1) de pueblos originarios.

443 participantes (54,8%, IC95%: 48,2-61,2) realizaron una consulta médica durante el año previo a la encuesta; 123 (15,4%, IC95%: 11,2-20,8) recurrieron a un servicio de urgencia dentro de los últimos 30 días. Frente a la necesidad de recurrir al médico por alguna emergencia de salud, 558 (69,1%, IC95%: 62,8-74,7) lo hicieron a un centro de salud, 165 (20,4%, IC95%: 15,7-26,1) a un hospital y 53 (6,5%, IC95%: 3,7-11,0) a un sanatorio privado. Con relación a los antecedentes cardiovasculares, 94 (11,6%, IC95%: 8,1-16,5) refirieron haber tenido algún evento cardíaco.

Para trasladarse a su lugar de trabajo la mayoría lo hacían caminando o a caballo [344 (71,8%), IC95%: 63,1-79,2], 113 (23,5%, IC95%: 16,6-32,2) utilizaban el automóvil y 19 (4,0%, IC95%: 1,8-8,9) bicicleta.

Respecto a los factores psicosociales, 218 (28,0%, IC95%: 22,8-33,9) dijeron sentirse sola/o a menudo, siendo un sentimiento significativamente mayor (p = 0,02) en las mujeres [132 (35,4%), IC95%: 28,5-43,0] comparado con los varones [86 (21,2%), IC95%: 14,2-30,4]. 374 participantes (46,3%, IC95%: 40,0-53,9) se sentían ansiosos o deprimidos y una gran mayoría [777 (96,2%), IC95%: 93,4-97,9] refirieron sentir estrés en el trabajo, hogar y/o por problemas económicos.

Las prevalencias por autorreporte crudas y ajustadas por edad para las diferentes variables se observan en la Tabla 3. Las prevalencias combinadas de DM, HTA y colesterol total superaron las obtenidas por autorreporte: DM 14,4% (IC95%: 10,8-18,8), HTA 41,9% (IC95%: 35,8-48,2), colesterol total 25,6% (IC95%: 20,8-31,1) (Figura 1). Hubo 22 participantes (6,3%) que, habiendo autorreportado no tener DM, tuvieron valores por encima del punto de corte de la normalidad en la etapa de mediciones físicas o bioquímicas; esta situación se observó en 66 participantes (17,1%) en el caso de la HTA y 87 (15,6%) para el colesterol elevado.

**Figura Nº 1.**
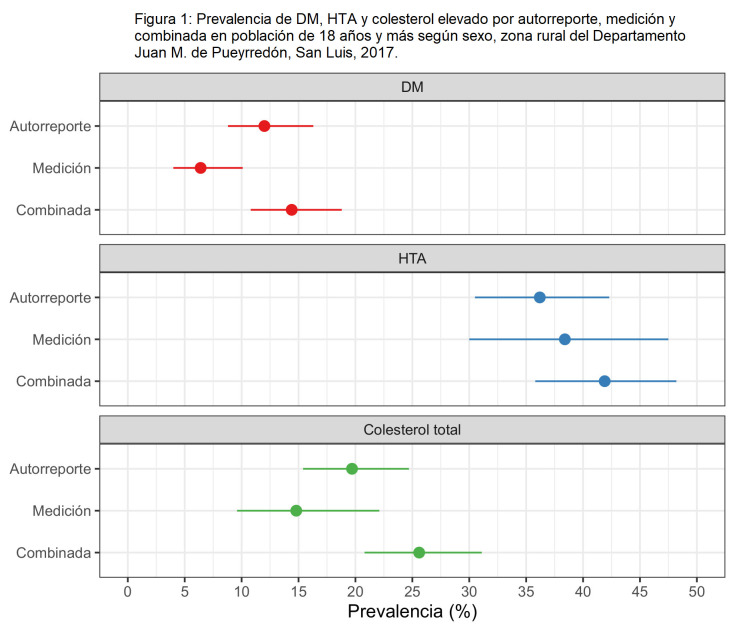
Prevalencia de DM, HTA y colesterol elevado por autorreporte, medición y combinada en población de 18 años y más según sexo, zona rural del Departamento Juan M. de Pueyrredón, San Luis, 2017.

**Tabla Nº 3:** Prevalencia cruda y ajustada de DM, HTA y colesterol elevado por autorreporte, en población de 18 años y más según sexo, zona rural del Departamento Juan M. de Pueyrredón, San Luis, 2017

	**Ambos sexos**		**Mujeres**		**Varones**	
**Variables**	**Prevalencia Cruda**	**Prevalencia ajustada**	**Prevalencia Cruda**	**Prevalencia ajustada**	**P** **revalencia Cruda**	**Prevalencia ajustada**
	**(IC95%)**	**(IC95%)**	**(IC95%)**	**(IC95%)**	**(IC95%)**	**(IC95%)**
DM	12,0 (8,8-16,3)	11,8 (8,2-15,4)	15,5 (10,6-22,0)	15,5 (9,8-21,1)	8,9 (5,1-15,1)*	8,8 (4,1-13,5)*
HTA	36,2 (30,5-42,3)	35,5 (31,0-40,1)	40,2 (33,1-47.8)	41,1 (34,0-48,3)	32,5 (24,3-42,0)	30,8 (24,0-37,6)
Colesterol elevado	19,7 (15,4-24,7)	20,3 (16,0-24,7)	21,3 (16,0-27,8)	22,7 (16,7-28,8)	18,2 (12,1-26,4)	18,3 (11,8-24,8)

344 participantes (42,6%, IC95%: 36,3-49,1) tenían antecedentes familiares de DM, sin diferencias según sexo. 52 personas que autorreportaron DM (53,8%, IC95%: 37,3-69,6) se encontraba bajo algún tipo de tratamiento, siendo mayoritario el farmacológico [38 (39,3%), IC95%: 24,8-56,0] y un poco más de la mitad controlaba su glucemia [50 (51,3%), IC95%: 35,0-67,2]. El 69,2% (IC95%: 59,9-78,4; N= 202) utilizaba algún tratamiento para su HTA; el 72,5% (IC95%: 63,0-82,1; N = 147) de ellos lo hacía sólo con medicamentos. El 56,5% (IC95%: 44,0-69,0; N= 90) recibía tratamiento para controlar los niveles de colesterol, en su mayoría con dieta (52,7%, IC95%: 34,7-70,6; N= 47).

Los varones tuvieron valores de colesterol HDL deseable y tensión arterial (TA) elevada en una proporción significativamente superior a las mujeres, mientras que estas últimas tuvieron obesidad abdominal en mayor magnitud (Tabla 4).

**Tabla Nº 4:** Distribución de variables construidas a partir de mediciones antropométricas y de laboratorio en población de 18 años y más según sexo, zona rural del Departamento Juan M. de Pueyrredón, San Luis, 2017.

**Variables**	**Ambos sexos (N = 808)**		**Mujeres (N = 384)**		**Varones (N = 424)**		p valor ^*^
	**Frec. abs.**	**Porcentaje (IC95%)**	**Frec. abs.**	**Porcentaje (IC95%)**	**Frec. abs.**	**Porcentaje (IC95%)**	
**Glucemia basal**							0,32
Normal	612	75,8 (67,5-82,5)	308	80,1 (71,3-86,8)	304	71,8 (57,7-82,6)	
Prediabetes	144	17,8 (11,7-26,1)	51	13,3 (7,7-21,9) ^a^	93	21,9 (12,1-36,4) ^a^	
DM	52	6,4 (4,0-10,1) ^a^	25	6,6 (3,5-11,9) ^a^	27	6,3 (3,1-12,3) ^a^	
**Hb1Ac (diag.)**							-
Normal	108	13,4 (8,0-21,6) ^a^	42	/// ^b^	66	/// ^b^	
Elevada	55	6,8 (4,2-10,9) ^a^	26	/// ^b^	29	/// ^b^	
Sin dato	645	79,8 (71,7-86,0)	316	82,3 (72,8-88,8)	329	77,6 (63,8-87,2)	
**Hb1Ac (control)**							-
Normal	128	15,9 (10,1-4,1) ^a^	50	13,2 (7,3-22,5)	78	/// ^b^	
Elevada	35	4,3 (2,5-7,3) ^a^	18	/// ^b^	17	/// ^b^	
Sin dato	645	79,8 (71,7-86,0)	316	82,2 (72,8-88,8)	329	77,6 (63,8-87,2)	
**Colesterol total**							
Normal	689	85,2 (77,9-90,4)	318	82,8 (74,3-89,0)	371	87,5 (73,7-94,5)	
Elevado	119	14,8 (9,6-22,1) ^a^	66	17,2 (11,0-25,7) ^a^	53	/// ^b^	
**LDL colesterol**							-
Normal	790	97,7 (95,3-99,0)	368	95,8 (90,9-98,3)	422	99,5 (96,2-99,9)	
Elevado	18	/// ^b^	16	/// ^b^	2	/// ^b^	
**HDL colesterol**							< 0,01
Normal	613	75,9 (66,6-83,2)	335	87,2 (77,6-93,1)	278	65,6 (50,5-78,0)	
Deseable	195	24,1 (16,8-33,4)	49	12,8 (6,9-22,4) ^a^	146	34,4 (22,0-49,5) ^a^	
Triglicéridos							0,76
Normal	697	86,3 (78,5-91,5)	335	87,3 (79,0-92,6)	362	85,3 (71,2-93,2)	
Elevado	111	13,7 (8,5-21,5) ^a^	49	12,7 (7,4-21,0)	62	/// ^b^	
**Tensión arterial**							< 0,05
Normal	497	61,5 (52,4-69,9)	271	70,6 (61,2-78,4)	226	53,3 (38,7-67,3)	
Elevada	310	38,4 (30,0-47,5)	112	29,1 (21,3-38,5)	198	46,7 (32,7-61,3)	
Sin dato	1	/// ^b^	1	/// ^b^	0	-	
**IMC**							0,19
Normal	208	25,7 (18,4-34,8)	88	22,9 (15,7-32,2)	120	28,3 (16,6-43,8) ^a^	
Sobrepeso	233	28,8 (21,3-37,8)	98	25,4 (17,8-34,8)	135	31,8 (19,9-47,2) ^a^	
Obesidad	354	43,8 (35,2-52,7)	197	51,4 (41,4-61,3)	157	37,0 (24,4-51,5)	
Sin dato	13	/// ^b^	1	/// ^b^	12	/// ^b^	
**Perímetro cintura**							< 0,01
Normal	371	45,9 (36,9-55,2)	87	22,6 (15,8-31,2)	284	67,0 (53,6-78,1)	
Obesidad abd.	424	52,5 (43,3-61,5)	296	77,1 (68,5-84,0)	128	30,2 (19,6-43,3)	
Sin dato	13	/// ^b^	1	/// ^b^	12	/// ^b^	

^*^ Test X^2^ con ajuste Rao & Scott

^a^ La estimación debe ser considerada con precaución por tener un CV entre 20 % y 30 %.

^b^ La estimación no es confiable ya que el CV es superior al 30%, la cantidad de casos involucrados es insuficiente o la proporción es menor al 5%.

Según el cuestionario FINDRISC, el 16,4% (IC95%: 11,0 - 23,6) ostentaba riesgo alto-muy alto de desarrollar DM2 en los próximos 10 años y el 21,4% (IC95%: 14,4 - 30,7) riesgo moderado (Figura 2).

**Figura Nº 2.**
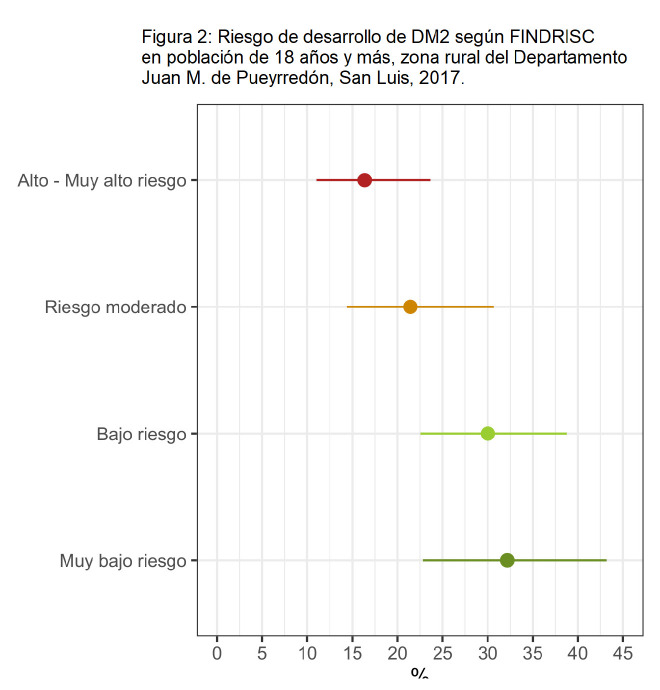
Riesgo de desarrollo de DM2 según FINDRISC en población de 18 años y más, zona rural del Departamento Juan M. de Pueyrredón, San Luis, 2017.

## Discusión

Nuestra investigación constituye un trabajo pionero en Argentina al caracterizar la situación de salud-enfermedad y condiciones de vida en una población rural, así como obtener estimaciones de prevalencia de ENT y factores de riesgo asociados. El conocimiento disponible en este campo proviene mayoritariamente de investigaciones llevadas a cabo en China^12^, India^13^ y Brasil(14,15).

La población estudiada tiene una distribución por sexo y edad similar a la de las áreas rurales de Argentina. El 4,9% nunca asistió a un establecimiento educativo; magnitud similar a la del país y superior a la de la provincia de San Luis (4,8% y 3,5% respectivamente)^8^.

Si bien la proporción de bajo nivel de actividad física en el tiempo libre fue alta en este trabajo, más del 75% utilizaba medios de transporte activos como caballo, bicicleta o caminata para dirigirse al trabajo. Esto va en el mismo sentido del perfil característico de los grupos rurales, más activos en los dominios desplazamiento y trabajo que en el tiempo libre, al igual que lo hallado en una investigación en comunidades rurales quilombolas de Brasil^15^. La estructura ocupacional de la población rural podría tener influencia en el nivel de actividad física que realizan, en comparación con las urbanas; el trabajo en áreas rurales implica tareas agrícolas y demás labores que requieren mayor vigor físico^16^.

Respecto del consumo de frutas y verduras, estudios realizados en Brasil alertaron sobre su bajo nivel en poblaciones rurales en comparación con las urbanas(17,18). Se atribuyó un rol preponderante a los cambios en los modos de producción de las últimas décadas, focalizados en el monocultivo y la exportación de sus productos, lo cual favorece la adopción de patrones alimentarios modernos ricos en grasas y carbohidratos simples(19,20). En Argentina se encontró que los hogares rurales tenían menor consumo aparente de hortalizas y frutas que los urbanos, por lo que se planteó el desafío de reforzar las estrategias de autoproducción de alimentos en las huertas familiares^5^. El consumo de frutas o verduras los siete días de la semana en nuestro trabajo fue significativamente mayor al hallado en un estudio previo de características similares a éste realizado en un aglomerado urbano de Argentina^21^ [45,8% (39,4-52,4) versus 36,3% (36,1-36,4)]. Sin embargo, nuestros resultados no nos habilitan a concluir sobre si estos niveles de consumo son óptimos debido a que la forma en que fue medido el evento no lo permite; en este sentido, estudios futuros que aborden esta dimensión con mayor detalle podrían aportar resultados más concluyentes.

Encontramos un alto porcentaje de participantes (96,5%) que refirieron sentir estrés a veces, con frecuencia o en forma permanente en el trabajo, el hogar o por motivos económicos; si bien dicha categoría conceptual resulta amplia en el sentido de contener una variedad de sentimientos posibles, llama la atención su magnitud cercana a la totalidad de la población estudiada. La proporción de personas con ansiedad o depresión fue superior a la reportada para localidades urbanas de Argentina^3^ y similar a la de un estudio en población rural de Portugal^22^.

Cabe subrayar el peso que tuvo el centro de salud a la hora de requerir consulta médica en esta población, señalado como lugar preferencial frente a emergencias médicas en el 69,1%; este perfil difiere del de un estudio en población mayoritariamente urbana de Argentina, que encontró que el lugar donde se acudía con mayor frecuencia ante la necesidad de atención fue el hospital^23^. Factores relacionados con la ubicación geográfica de las comunidades rurales y la concentración de servicios hospitalarios en centros urbanos podrían explicar esto; una revisión sistemática sobre el tema demostró que dichos aspectos interferían con la capacidad de las poblaciones rurales de acceder a la atención de salud de manera oportuna, favoreciendo la búsqueda de servicios de salud en la propia comunidad^4^En cuanto a la magnitud de la DM, la referencia internacional proviene de la Federación Internacional de Diabetes^24^ que estimó una prevalencia cruda de 7,2% para la población rural a nivel global. En nuestro trabajo hallamos una prevalencia cruda por autorreporte superior, sin poder concluir sobre la significancia estadística de tales diferencias. La prevalencia que obtuvimos fue mayor, aunque no significativamente, a la calculada en comunidades rurales del Estado de Goiás, Brasil^16^. A nivel nacional, en comparación con las prevalencias ajustadas por edad (por autorreporte) calculadas a partir de los datos de la ENFR 2018^3^, la prevalencia de DM en nuestra investigación fue similar a la de Argentina e inferior a la de la provincia de San Luis [11,8% (IC95%: 8,2-15,4) vs 12,0% (IC95%: 11,4-12,7) y 16,8% (IC95%: 13,3-20,2) respectivamente]. La prevalencia de HTA fue 2,3% mayor a la calculada para el país y 4,2% inferior a la de San Luis [35,5%, IC95%: 31,0-40,1] vs 33,2% (IC95% > 32,2-34,1) y 39,7% (IC95%: 35,1-44,3) respectivamente; las diferencias encontradas no fueron estadísticamente significativas. Hallamos una prevalencia de colesterol elevado significativamente más baja que la de Argentina y más aún que a nivel provincial [20,3% (IC95%: 16,0-24,7) vs 28,3% (IC95%: 27,0-29,5) y 33,3% (IC95%: 29,0-37,6) respectivamente]. No encontramos diferenciales por sexo en las prevalencias de DM, HTA y colesterol elevado. Los niveles de sobrepeso y obesidad no tuvieron diferencias significativas con lo registrado a nivel nacional para áreas urbanas^3^.

Un aspecto llamativo en nuestro trabajo fue la alta proporción de personas que no atendían sus problemas de salud como DM, HTA e hipercolesterolemia; prácticamente la mitad de los que tenían diagnóstico de DM o colesterol elevado no se trataban. La magnitud fue mayor a la encontrada en el aglomerado urbano Mar del Plata-Batán de Argentina^21^ y similar a la de la población urbana de Argentina^8^; este problema ha sido puesto de manifiesto asimismo por investigaciones en otros países, tanto en población rural como urbana(25,26). La relevancia de esta cuestión en el campo de las ENT se ve reflejada en el concepto de carga del tratamiento y sus consecuencias en la vida cotidiana de las personas, las familias y el sistema de salud^27^. Consideramos que este asunto amerita la realización de nuevas investigaciones, con metodologías cualitativas, que posibiliten avanzar en su comprensión y abordaje.

Nuestra estimación del nivel de riesgo de desarrollar DM2 en 10 años alto-muy alto fue similar a lo observado en una comunidad rural de Venezuela^28^ y al de la población urbana de Argentina^8^.

Con relación a los diferenciales por sexo en las características sociodemográficas, las mujeres ostentaron mayor nivel educativo, en consonancia con el patrón de las poblaciones rurales y urbanas tanto de la provincia de San Luis como de Argentina en su conjunto^8^. La condición de encontrarse sin trabajo, más frecuente en las mujeres, fue en el mismo sentido que lo encontrado en el aglomerado urbano Mar del Plata-Batán^21^. La menor magnitud del hábito tabáquico actual y el bajo nivel de actividad física de las mujeres fue similar a lo observado en población urbana a nivel nacional. Una investigación en comunidades rurales indígenas de las islas Fiji mostró que los hombres enfatizaron los deportes y el trabajo en las tierras de cultivo como tipos preferidos de actividad física, mientras que las mujeres se enfocaron en actividades familiares, diarias y de apoyo para áreas de juego específicas para mujeres, reforzando los roles tradicionales de género(29,30).

Entre las debilidades de nuestra investigación, señalamos algunas cuestiones metodológicas que deben tenerse en cuenta en la interpretación de sus resultados. La tasa de respuesta obtenida alcanzó en general niveles aceptables, con excepción de la localidad de Cazador. El autorreporte puede traer aparejada una sub o sobreestimación del parámetro según el dominio evaluado. Sin embargo, su buena sensibilidad avala su utilización en el campo de las ENT.

Como fortalezas, destacamos la contribución pionera de este trabajo al conocimiento del perfil de salud-enfermedad de las comunidades rurales de Argentina, las cuales escapan habitualmente de los objetivos de la vigilancia de ENT, constituyendo un insumo de valor primordial en la planificación de políticas específicas para la población rural.
